# Usage of a rotational flap for coverage of a large central forehead defect

**DOI:** 10.3205/iprs000104

**Published:** 2017-02-07

**Authors:** Ahmed Hassan El-Sabbagh

**Affiliations:** 1 Plastic Surgery Center, Faculty of Medicine, Mansoura University, Mansoura, Egypt

**Keywords:** forehead, large defect, central area, rotation flap

## Abstract

**Background:** The forehead is a donor site for facial reconstruction but has no generous donor site for its coverage. All options of the reconstructive ladder can be used. A large rotation flap was used to reconstruct a big central forehead defect following failed previous repair in an elderly diabetic patient after a motor car accident.

**Case presentation:** A 64-year-old diabetic man presented with an extensive central forehead defect after failed previous repair following a motor car accident. Coverage of the defect was performed using a flap based around the ear on one side in a rotation movement. An accepted functional and esthetic result was achieved after 3 months of follow-up.

**Conclusion:** A rotation flap based on arteries around the ear can be used for coverage of a difficult lesion in the central forehead.

**Level of evidence:** Level V, therapeutic study

## Background

The forehead is a donor site for facial reconstruction but has no generous donor site for its coverage. All options of the reconstructive ladder can be used [1]. Recently dermal substitute (Integra) and VAC therapy were added as reconstructive methods for forehead reconstruction [2], [3]. The selected method of reconstruction is governed by the size and the site of the lesion, the exposed structures, and the general condition of the patient.

A large rotation flap was used to reconstruct a large central forehead defect following failed previous repair in an old diabetic patient after a motor car accident. 

## Method

The scalp is divided into four vascular territories. They interconnect to form an extensive network covering the entire scalp. The anterior territory is supplied by the supraorbital and the supratrochlear arteries. The lateral territory is supplied by the superficial temporal artery. It is the longest scalp vessel and supplies the largest area. The posterolateral territory is vascularized by the posterior auricular artery and its branches. They are tightly stuck to the mastoid process of the temporal bone. The posterior scalp territory is supplied by the two lateral and the two medial occipital artery branches. Below the nuchal line, multiple small perforators from the trapezius and splenius muscles supply the scalp [4].

Miller and his colleagues performed 13 vascular anastomoses for the first replanted scalp [5]. Recent studies showed that the angiosomes anastomose in the scalp with one another on each side and across the midline. Some connections are significant “choke” anastomoses [6]. So it has been possible to replant the entire scalp with one arterial and one venous anastomosis [7].

### Rotation flap

The rotation flap is semicircular in shape and rotates around a pivot point to the defect. To facilitate its movement along its arc, a back-cut can be done or Burow’s triangle can be removed externally to the pivot point. The donor site can be closed primarily or by a skin graft.

## Case presentation

**Figure 1:**
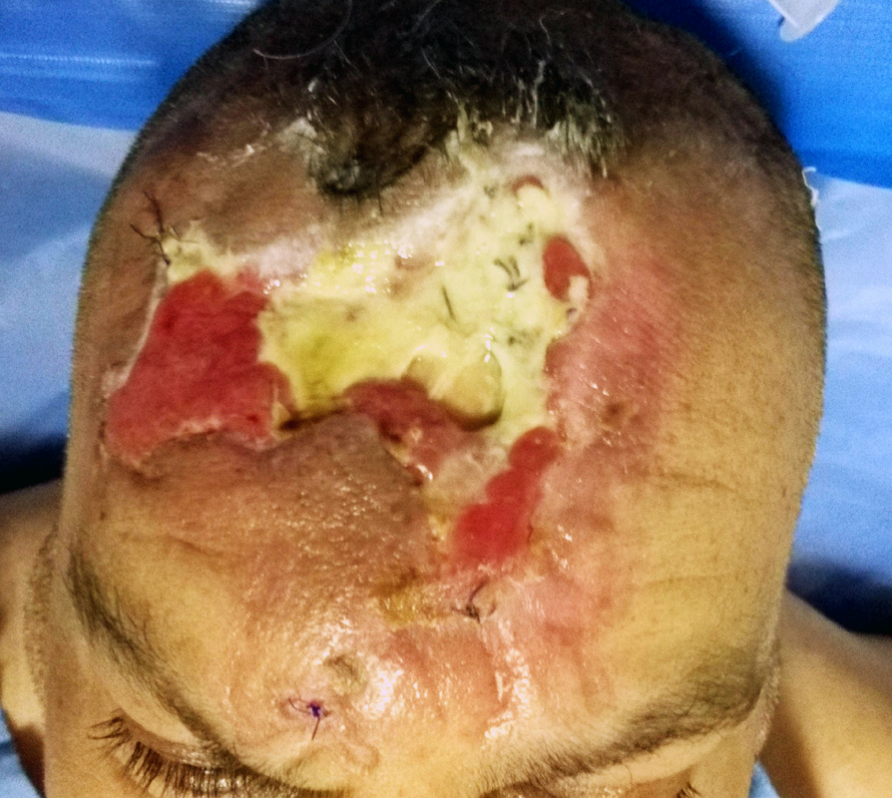
The defect in the central area of the forehead with signs of previous repair associated with bone exposure

A 64-year-old man, known for diabetes managed with insulin therapy since 20 years, had a motor car accident leading to a degloving injury and a skin maceration of his central forehead. It was repaired by local flaps in a private hospital. As a complication, he developed a skin necrosis with exposure of bone. The defect size was 17x10 cm. Six weeks after the previous repair he was presented to our center (Figure 1 (Fig. 1)).

Considering the importance of a reliable coverage after bone debridement and the relative medical condition of the patient precluding a free flap or multiple stages operations, a reconstruction by a big rotation flap was planned.

Because of the patient’s systemic fragility, hospitalization with tight control of blood sugar was done 3 days before the operation. The most suitable vessels for the flap were the superficial temporal artery and the postauricular artery on the left side. The arteries were checked with Doppler probe preoperatively.

**Figure 2:**
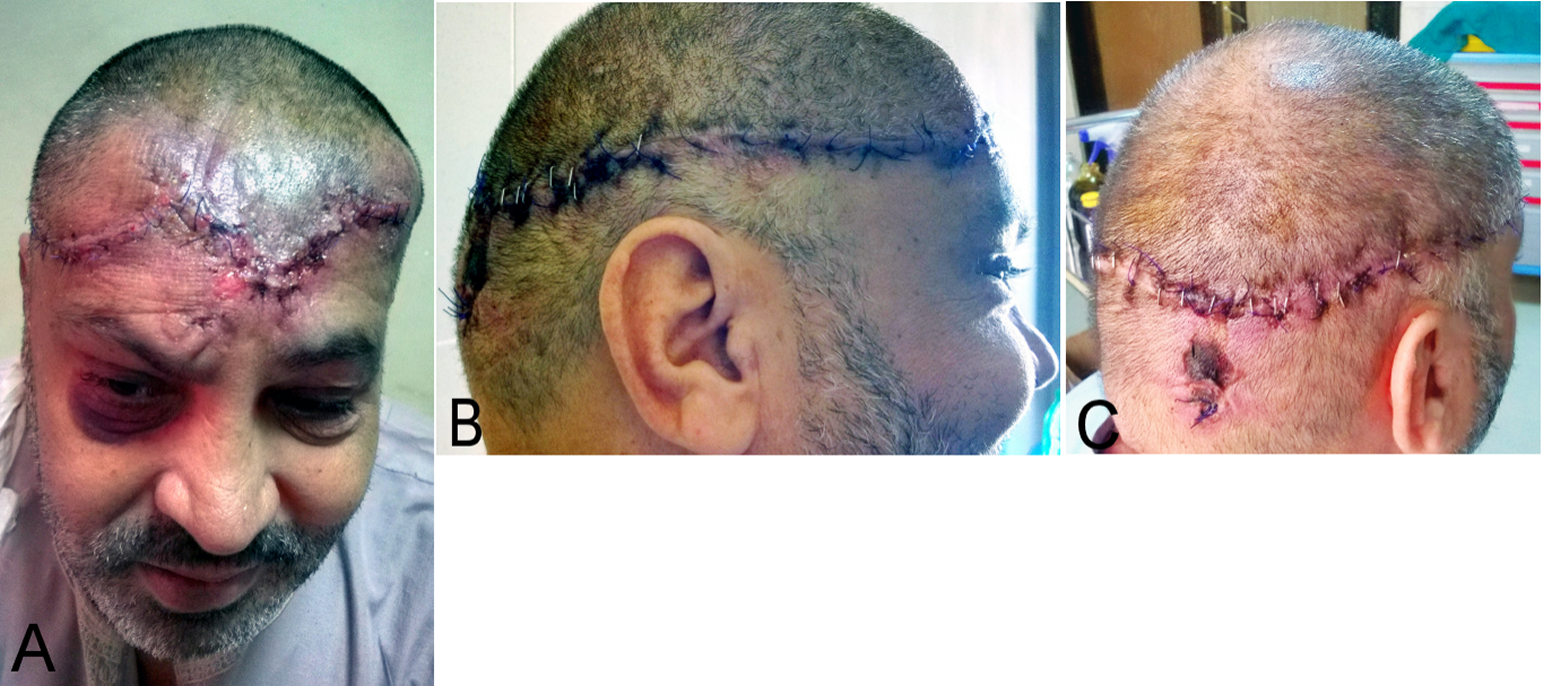
The course of the incision line of the flap. A: anteriorly, B: laterally, C: posteriorly.

After debridement and removal of all necrotic tissues and cleaning of bone, the scalp was infiltrated with tumescent solution along the proposed incision line. The incision line was started from the largest side of the defect (right side). Anteriorly, the incision was extended along the frontal hairline. As the incision was going laterally on the right side, the flap was widened up to 2 cm above the hairline. Posteriorly, the incision was lowered down to the middle of the occipital area (Figure 2 (Fig. 2)).

**Figure 3:**
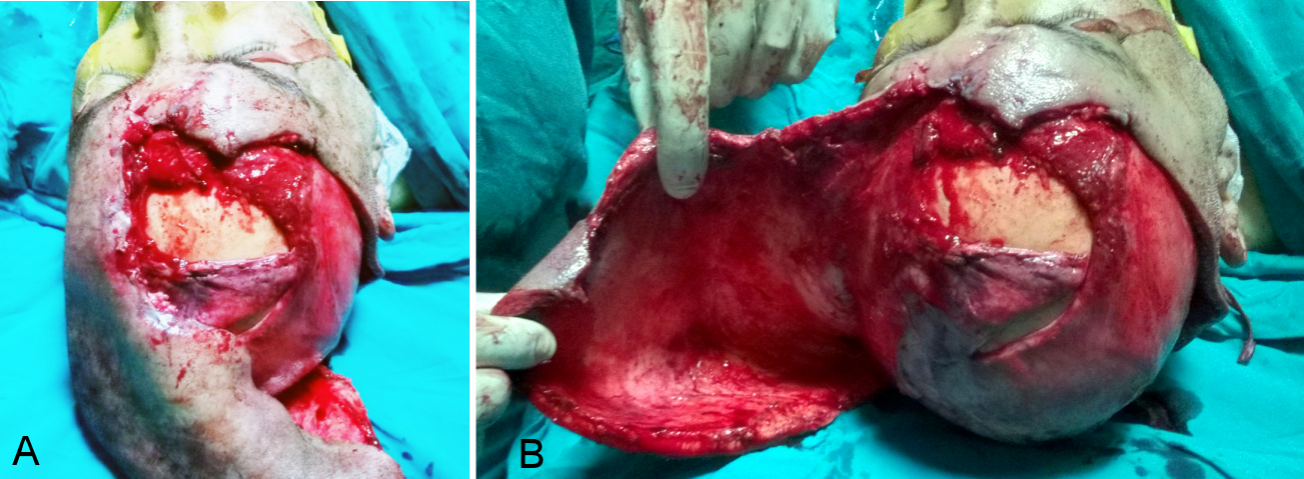
Dissection of the flap around the ear. A: the arc of the flap, B: the undersurface of the dissected area of the flap.

The incision was stopped before approaching the left ear. Then, the flap was dissected anteriorly and laterally on the left side without injury to the feeding vessels (Figure 3 (Fig. 3)). The flap was based on the following arteries: superficial temporal and postauricular arteries. The arteries were checked by intraoperative Doppler to ensure the integrity of the feeding vessels.

Careful hemostasis was done and a drain was inserted. The wound was dressed and light compression applied over it.

### Postoperative care

**Figure 4:**
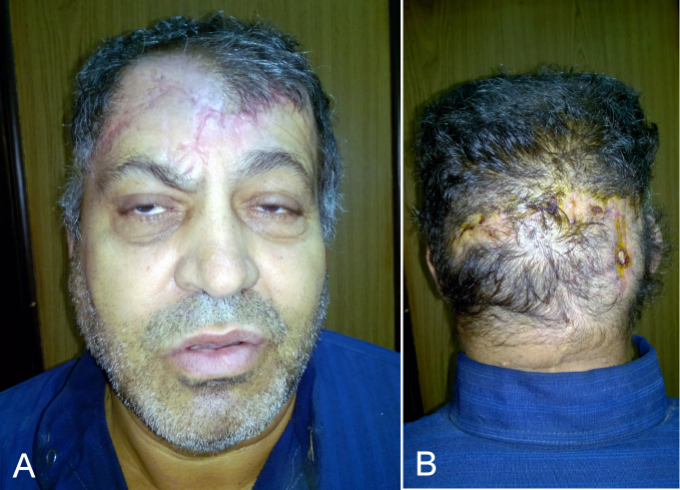
Postoperative results after 3 months. A: anterior view, B: posterior view.

The dressing was changed on the 1^st^ postoperative day and a light compression was reapplied again. The tip of the flap was exposed to check the vascularity of the flap. Blood sugar was monitored carefully. The drain was removed after 10 days and healing was excellent. Follow-up was extended up to 3 months (Figure 4 (Fig. 4)).

## Discussion

The aesthetic significance of the forehead and the scantiness of loose donor tissue can present a great challenge for reconstructive surgeons. Primary closure is an ideal solution for defects less than 3 cm in size [8]. Bilateral advancement flap (H-plasty) was described for coverage of small- to medium-sized defects [9]. In this patient, the defect was too large to be closed with H-plasty technique.

When dealing with larger defects, several methods of reconstruction are used. Skin grafts offer adequate coverage for larger defects. The wound bed is allowed to granulate for at least 3 weeks before application of skin grafts. Exposure of bone combined with unhealthy bed precluded the use of grafting technique in this patient. Generally speaking, skin grafts on the forehead are avoided. They have several disadvantages such as color mismatching, contour irregularities and cannot cover vital structures [10], [11]. 

Tissue expansion can be used to achieve aesthetically pleasing results. In this case, this technique was not ideal because there was an increased risk of infection due to diabetes and old age. Also, a long time was needed for this defect. Additionally, two stages were required [12], [13], [14], [15]. 

Free flaps are ideal for defects exceeding 50 cm^2^, with plenty of different components that can be included in the flap [16], [17], [18], [19], [20]. However, it is often complicated with partial or total flap loss, bulky in size and a lengthy recovery period with high costs is needed [21].

Actually, local flaps provide like tissue for coverage, but they had previously been applied for large defects resulting in multiple scarring and secondary defects covered by skin grafts [22], [23].

In the 1940s, Converse designed a scalp flap for nasal reconstruction. Its blood supply was based as forehead flap [24]. Then, three flaps were designed for scalp defects but were not preferable for forehead reconstruction [25]. 

In this case, there was a large sized (15x10 cm) and challenging defect, including skin necrosis and bone exposure. The flap was designed as a single broad flap for a large central forehead defect. Soft tissue coverage and healing was uneventful and the donor site was closed primarily. The frontal branch of the facial nerve was evaluated (see video in Attachment 1). Also the level of eyebrows was not elevated on the incision site (right side). 

In summary, the placement of the anterior incision on the frontal hairline towards the occipital area prevents to a great extent the elevation of the eyebrow, preserves the hairline, and avoids the injury of the frontal branch of the facial nerve. A drawback to consider is the long scar of the flap which may limit its use in patients with high aesthetic demands. In our case, the patient was elderly and recovered from a previous failed operation. 

This flap presents many advantages in the central area of the forehead such as being a single flap with a reliable blood supply. Because of the robust collateral circulation, a single arterial blood supply is sufficient to maintain vascularity of the scalp [26]. Also, this flap is an alternative to free flaps, avoiding the need for microsurgical anastomosis, and reducing the operating time. Also, the option of using tissue expander was discarded, eliminating the risk of infection, exposure of prosthesis, and two stage procedures. Donor site morbidity was minimal as there was no secondary defect and no tedious dissection. 

## Conclusions

A rotation flap based on arteries around the ear can be used for coverage of a difficult lesion in the central forehead. Replacing ‘like with like’ allows for nice texture and good esthetic results.

## Notes

### Competing interests

The authors declare that they have no competing interests.

### Ethical standards

This study has been performed in accordance with the ethical standards set forth in the 1964 Declaration of Helsinki and its later amendments. Patient consent for the publication of the photographs and the video was obtained.

## Supplementary Material

Video: Evaluation of the frontal branch of the facial nerve
